# Protective Effect of Low Molecular Weight Seleno-Aminopolysaccharide on the Intestinal Mucosal Oxidative Damage

**DOI:** 10.3390/md17010064

**Published:** 2019-01-18

**Authors:** Zheng-Shun Wen, Zhen Tang, Li Ma, Tian-Long Zhu, You-Ming Wang, Xing-Wei Xiang, Bin Zheng

**Affiliations:** 1 Zhejiang Provincial Engineering Technology Research Center of Marine Biomedical Products, School of Food Science and Pharmaceutics, Zhejiang Ocean University, Zhoushan 316022, China; tz19920904@126.com (Z.T.); 15150512714@163.com (L.M.); 6369958@163.com (B.Z.); 2 Department of Agriculture, Jiaxing Vocational Technical College, Jiaxing 314036, China; tianlong4183@163.com; 3 Key Laboratory of Animal Nutrition and Feed Science in East China, Ministry of Agriculture, College of Animal Science, Zhejiang University, 866 Yuhangtang Road, Hangzhou 310058, China; 4 Zhejiang Marine Development Research Institute, Zhoushan 316021, China

**Keywords:** low molecular seleno-aminopolysaccharide, intestinal mucosa, oxidative damage, Nrf2–Keap1 pathway

## Abstract

Low molecular weight seleno-aminopolysaccharide (LSA) is an organic selenium compound comprising selenium and low molecular weight aminopolysaccharide (LA), a low molecular weight natural linear polysaccharide derived from chitosan. LSA has been found to exert strong pharmacological activity. In this study, we aimed to investigate the protective effect of LSA on intestinal mucosal oxidative stress in a weaning piglet model by detecting the growth performance, intestinal mucosal structure, antioxidant indices, and expression level of intracellular transcription factor nuclear factor erythroid 2-related factor 2 (Nrf2) and its related factors. Our results indicated that LSA significantly increased the average daily gain and feed/gain (*p* < 0.05), suggesting that LSA can effectively promote the growth of weaning piglets. The results of scanning electron microscope (SEM) microscopy showed that LSA effectively reduced intestinal damage, indicating that LSA improved the intestinal stress response and protected the intestinal structure integrity. In addition, diamine oxidase (DAO) and d-lactic acid (d-LA) levels remarkably decreased in LSA group compared with control group (*p* < 0.05), suggesting that LSA alleviated the damage and permeability of weaning piglets. LSA significantly increased superoxide dismutase (SOD), catalase (CAT), glutathione peroxidase (GSH-Px), and total antioxidant capacity (T-AOC) levels, but decreased malondialdehyde (MDA) level, indicating that LSA significantly enhanced the antioxidant capacity and reduced oxidative stress in weaning piglets. RT-PCR results showed that LSA significantly increased GSH-Px1, GSH-Px2, SOD-1, SOD-2, CAT, Nrf2, HO-1, and NQO1 gene expression (*p* < 0.05). Western blot analysis revealed that LSA activated the Nrf2 signaling pathway by downregulating the expression of Keap1 and upregulating the expression of Nrf2 to protect intestinal mucosa against oxidative stress. Collectively, LSA reduced intestinal mucosal damage induced by oxidative stress via Nrf2-Keap1 pathway in weaning stress of infants.

## 1. Introduction

The clinical onset and severity of intestinal disorders can be markedly impacted by early life stress in humans and animals [1]. In early life, human infants and neonatal piglets experience significant stress, which are associated with homeostatic changes related to birth and weaning. Adaptive changes to these early-life stressors are likely contributors to future gastrointestinal disease susceptibility [2]. Weaning stress results in intestinal mucosal oxidative damage, increases intestinal mucosal damage, and endangers the health of the body. Therefore, maintaining the healthy state of intestinal mucosa during weaning has become a research hotspot. Increasing attention has been paid to large animal models, especially pigs, as an important translational model for the study of human gastrointestinal disease owing to their remarkably similar gastrointestinal physiology and anatomy [3,4]. We used piglets as an animal model in our previous study to simulate the function of intestinal barrier of human infants during oxidative stress induced by weaning [5].

Oxidative stress is one of the main factors causing various diseases in humans and animals, especially intestinal diseases [6]. Being the largest contact surface between the body and the outside world, the intestine is the central organ of stress response when the body is under stress [7]. The gastrointestinal tract is easily attacked by excessive free radicals, resulting in abnormal metabolism of intestinal epithelial cells to be blocked, impaired cell function, and inflammatory reaction [8]. Moreover, oxidative stress damages intestinal mucosal morphology and increases permeability, intestinal mucosal immune dysfunction, and intestinal disease [9,10]. Oxygen free radicals can result in DNA damage, protein degeneration, lipid peroxidation, signal transduction abnormalities, energy metabolism disorders, mitochondrial damage, and apoptosis in the intestinal tract [11]. Thus, oxidative stress causes inflammatory bowel disease, intestinal mucosal infection, Crohn’s disease, ulcerative colitis, and colon cancer [12]. 

Organic selenium is an important selenium sources containing selenium amino acid, selenium yeast, and selenium polysaccharide. Selenium polysaccharide is the most common among them. A previous study reported that selenium polysaccharide had higher or unique biological activity than simple superposition of selenium and polysaccharide activity [13]. However, the purity of selenium polysaccharides is low in nature. Therefore, increasing attention has been paid to selenium polysaccharides synthesis [14]. Chitosan is non-toxic (LD_50_ >16 g/kg), and non-immunogenic, biodegradable, and can be manufactured reproducibly based on good manufacturing practice (GMP) guidelines [15]. Low molecular weight aminopolysaccharide (LA), which is a derivative of chitosan obtained from the deacetylation of chitin from crustaceans, insects, and fungi, is an abundant, natural linear polysaccharide. LA has been reported to exert many biological activities, including antioxidant and antitumor activities. Our previous studies suggested that low molecular weight seleno-aminopolysaccharide (LSA) had lower acute toxicity than sodium selenite, and higher antioxidant and immunomodulatory activities than selenium and/or LA alone [5,16,17]. Nevertheless, few studies have investigated the effect of seleno-aminopolysaccharide on the intestinal mucosal oxidative damage induced by weaning stress. Therefore, our laboratory used LA and sodium selenite to synthesize LSA. In the present study, an animal model of intestinal mucosal oxidative injury induced by weaning stress was used to investigate the protective effects of LSA on intestinal mucosal and to elucidate the possible mechanism of action. The present study may provide novel evidence to understand the potential mechanism underlying the protective effects of LSA on the intestinal mucosal injury, which might be a better alternative agent for the prevention and treatment of weaning stress in infants. 

## 2. Results

### 2.1. Effects of LSA on Growth Performance

Effects of LSA on growth performance in weaning piglets are shown as Table 1. The results showed that LSA (0.3 and 0.6 mg/kg) significantly enhanced the daily gain of weaning piglets and reduced the feed/gain ratio (*p* < 0.05). However, no significant difference was found among the sodium selenite group (S), sodium selenite + LA group (S+) and control group (C). The difference in feed intake between the groups was not significant (*p* > 0.05). The results indicated that LSA can effectively promote the growth of weaning piglets and has good growth performance, which may be related to the reduction of weaning stress in piglets in LSA groups.

### 2.2. Effects of LSA on the Diarrhea Rate

**Figure 1:**
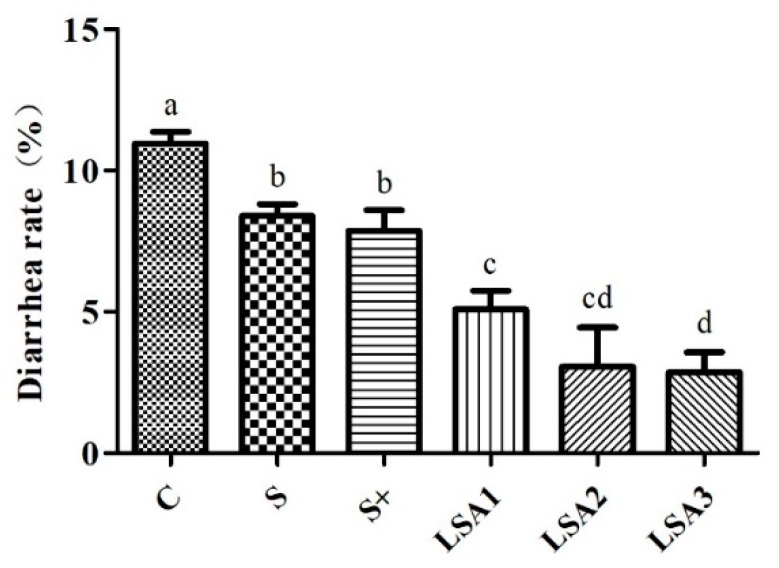
Effects of LSA on diarrhea rate. Bars labeled with different letters (a–d) were significantly different (*p* < 0.05) from each other.

Effects of LSA on the diarrhea rate are shown in Figure 1. The results have shown that LSA significantly reduced the diarrhea rate of weaning piglets compared with sodium selenite group and sodium selenite + LA group (*p* < 0.05). This indicates that LSA can effectively control weaning stress-induced diarrhea, which may reduce the stress response in the intestines and show a significantly protective effect on the intestines of weaning piglets.

### 2.3. Effects of LSA on Intestinal Morphology

**Figure 2:**
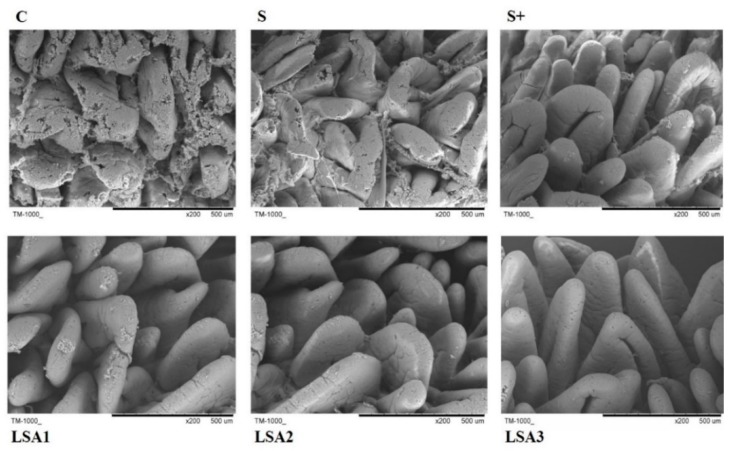
Effects of LSA on intestinal morphology (SEM, 200×).

The effects of LSA on intestinal morphology are shown in Figure 2. Weaning significantly induced injury of the intestine in piglets. Intestinal mucosal damage was reduced to a certain extent in sodium selenite group and sodium selenite + LA group, but the effect was weaker than that in LSA groups. Scanning electron microscopy (SEM) images showed that LSA effectively reduced intestinal damage, suggesting that LSA could significantly improve the intestinal stress response induced by weaning and protect the intestinal structure integrity of piglets.

### 2.4. Effects of LSA on the Level of DAO, d-LA in Serum

**Figure 3:**
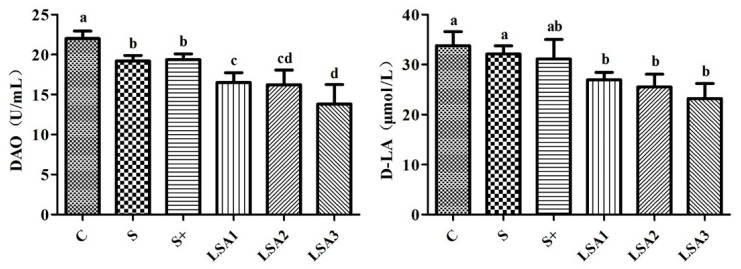
Effects of LSA on the level of diamine oxidase (DAO) and d-lactic acid (d-LA) in serum. Bars labeled with different letters (a–d) were significantly different (*p* < 0.05) from each other.

As shown in Figure 3, weaning induced higher serum levels of diamine oxidase (DAO) and d-lactic acid (d-LA). LSA significantly reduced DAO and d-LA levels. Sodium selenite group and sodium selenite + LA group also significantly reduced the DAO level compared with the control. However, no significant difference was found in reduction of d-LA level. In addition, LSA could significantly reduce DAO and d-LA levels compared with sodium selenite group and sodium selenite + LA group (*p* < 0.05). These results suggested that LSA significantly reduced the intestinal damage and permeability. 

### 2.5. Effects of LSA on the Antioxidant Levels in Serum

**Figure 4:**
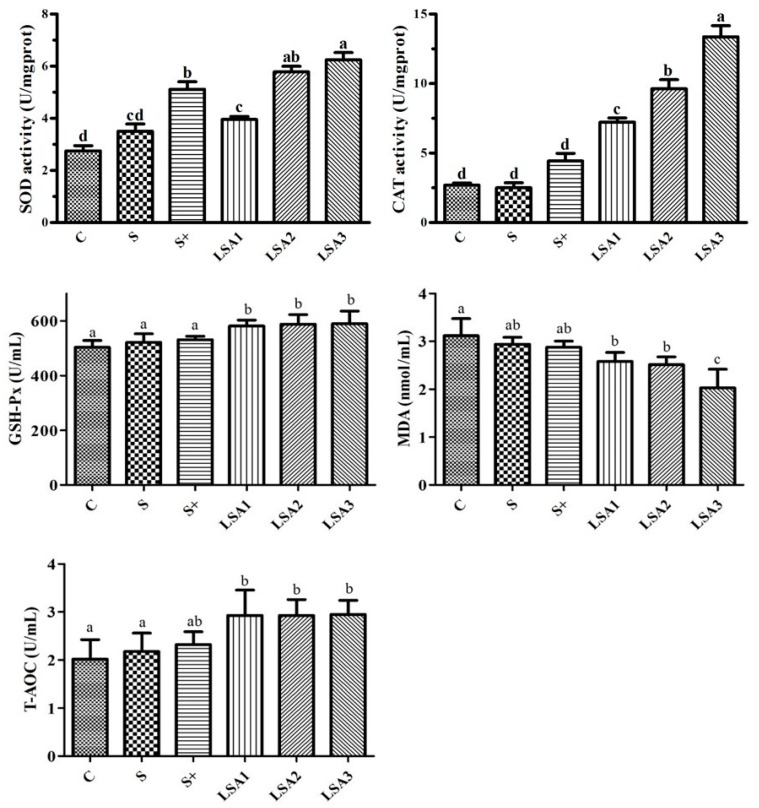
Effects of LSA on the antioxidant indices in serum. Bars labeled with different letters (a–d) were significantly different (*p* < 0.05) from each other.

Effects of LSA on the antioxidant levels of weaning piglets are shown in Figure 4. Compared with the control group, LSA were significantly increased superoxide dismutase (SOD), catalase (CAT), glutathione peroxidase (GSH-Px), and total antioxidant capacity (T-AOC) levels, and significantly decreased the malondialdehyde (MDA) level (*p* < 0.05). Sodium selenite group and sodium selenite + LA group also enhanced the level of SOD, CAT, GSH-Px, and T-AOC, but there was no significant difference. These results were indicated that LSA significantly enhanced the antioxidant capacity and reduced oxidative stress in weaning piglets, and its antioxidant effect was better than that of sodium selenite group and sodium selenite + LA group.

### 2.6. Effect of LSA on the Gene Expression Levels of Tight Junction Proteins in the Ileum

**Figure 5:**
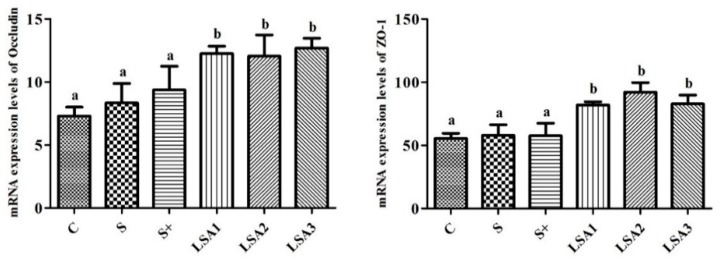
Effect of LSA on the gene expression levels of tight junction proteins in ileum. Bars labeled with different letters (a, b) were significantly different (*p* < 0.05) from each other.

The effects of LSA on the gene expression level of tight junction protein (ZO-1 and Occludin) in the ileum are shown in Figure 5. LSA significantly enhanced the gene expression levels of ZO-1 and Occludin in ileal mucosa compared with the control, sodium selenite group and sodium selenite + LA group (*p* < 0.05). These findings suggested that LSA effectively protected the ileum tight junction, reduced the permeability of the intestinal tract, and enhanced the barrier function.

### 2.7. Effects of LSA on the Expression of Antioxidant Genes in the Ileum

**Figure 6:**
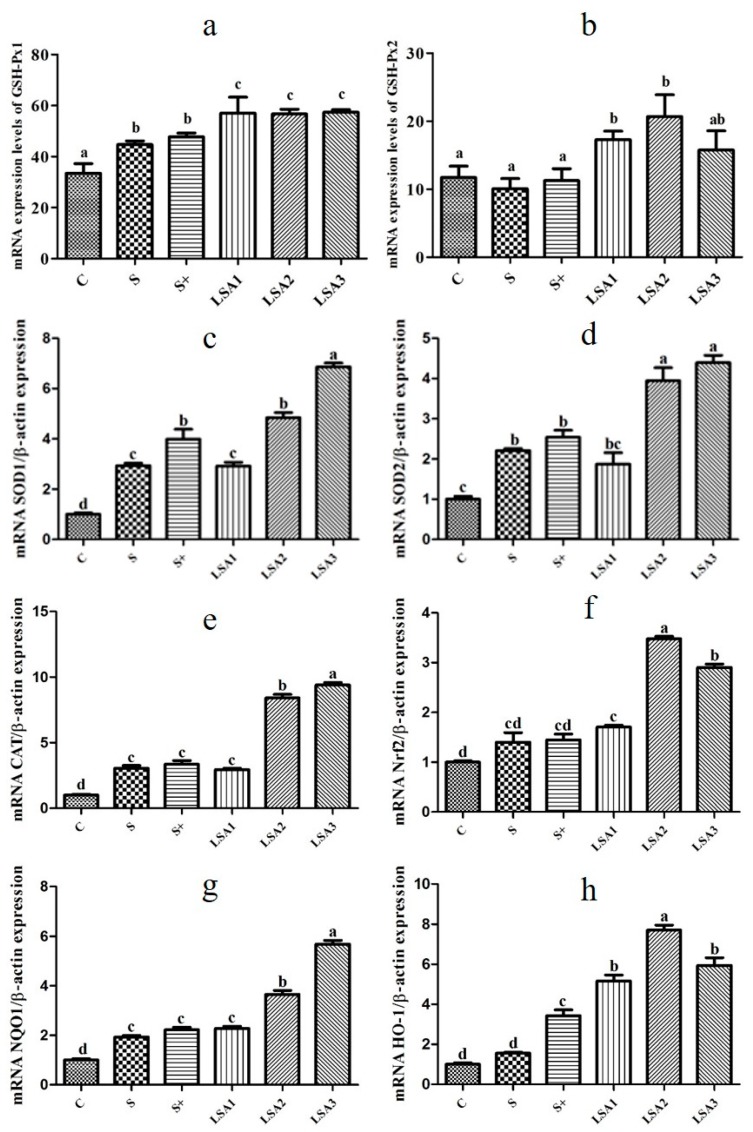
Effects of LSA on the expression of antioxidant genes in ileum. The mRNA expression level of antioxidant genes (GSH-Px1 (**a**), GSH-Px2 (**b**), SOD1 (**c**), SOD2 (**d**), CAT (**e**), Nrf2 (**f**), NQO1 (**g**), HO-1 (**h**). Bars labeled with different letters (a–d) were significantly different (*p* < 0.05) from each other.

As shown in Figure 6, LSA remarkably enhanced the expression level of antioxidant genes (GSH-Px1/2, SOD1/2, CAT, Nrf2, NQO1, HO-1) compared with the other groups. It can be suggested that LSA significantly enhanced the intestinal antioxidant capacity and reduced intestinal oxidative damage caused by weaning stress.

### 2.8. Effects of LSA on Expression Level of Kelch-Like ECH-Associated Protein 1 (Keap1) and Nrf2

**Figure 7:**
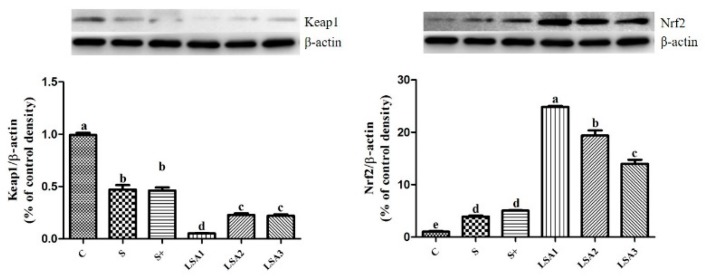
Effects of LSA on the Keap1–Nrf2 signaling pathway in ileum. Bars labeled with different letters (a-d) were significantly different (*p* < 0.05) from each other.

The effects of LSA on the expression level of intestinal Nrf2 are shown in Figure 7. The experimental groups remarkably decreased the expression level of Keap1 protein and significantly enhanced the expression level of Nrf2 protein (*p* < 0.05) compared with the control. In addition, LSA has significantly inhibited Keap1 protein expression and improved Nrf2 protein expression compared with that of sodium selenite group and sodium selenite + LA group.

## 3. Discussion

Under normal physiological conditions, the intestine is not only an important barrier that prevents harmful substances (bacteria and toxins) from invading the body, but also an important organ that regulates the stress response of the body and produces inflammatory mediators [18,19]. As the largest component of the immune system, the intestine is continually attacked by excessive free radicals leading to oxidative stress which, in turn, results in intestinal cell DNA damage, protein denaturation, lipid peroxidation, abnormal signal transduction, and apoptosis [11,20,21]. These mechanisms are responsible for the occurrence and development of diarrhea, intestinal mucosal infection, intestinal inflammatory disease, and colon cancer [12,22]. Therefore, the function of the intestine plays an important role in maintaining the overall health of the body. Intestinal dysfunction can cause a range of diseases. Among them, diarrhea is the most common phenomenon, especially in early weaning infants. Mild diarrhea can lead to malnutrition and growth retardation, while severe diarrhea may lead to dehydration and even death. Previous studies demonstrated that organic selenium significantly reduced the diarrhea incidence and improved the immune system function and growth performance [23,24]. Moreover, some studies revealed that organic selenium and inorganic selenium could affect the growth performance to a certain extent, however, selenium exerted better effects than inorganic selenium [25,26,27]. In the present study, results showed that LSA significantly enhanced the average daily gain, improved the feed efficiency, and effectively promoted the growth of weaning piglets. In addition, compared with the inorganic selenium group, LSA significantly reduced the diarrhea rate, suggesting that LSA effectively controlled diarrhea and reduced the intestinal stress response in weaning piglets. Thus, LSA was found to improve the growth performance of piglets, which was attributed to the protective effect on intestinal mucosa.

Destruction of the intestinal mucosa can result in various intestinal-derived diseases [28]. The tight junction between intestinal mucosal epithelial cells are the primary means of cell to cell connection [29]. The tight junction molecules comprise three membrane protein Occludins, and junctive adhesion molecules (JAMs) and zonula occludens (ZO) families. The tight junction proteins are important indicators of observing intestinal tight junction barrier and permeability function. Among them, ZO-1 and Occludin are important proteins that are tightly linked to the expression of barrier function, which plays a key role in maintaining the integrity of tight junctions [30,31]. Some reports demonstrated that weaning is related to increased intestinal permeability and inflammation, villous atrophy of the small intestine, and reduced immunological response [32,33,34]. Therefore, elucidation of changes in the expression of tight junction molecules is important for understanding intestinal mucosal barrier function, and for preventing and treating certain diseases. In this study, the mRNA expression levels of ZO-1 and Occludin significantly decreased in the weaning group, which indicated an increase in intestinal permeability. However, LSA significantly enhanced the expression level of ileum mucosa connexin gene ZO-1 and Occludin in ileum mucosa compared to the weaning group. In addition, intestinal mucosal integrity was observed by measuring the levels of DAO and d-LA. In this study, LSA significantly decreased DAO and d-LA level, suggesting that LSA reduced the intestinal damage and permeability of weaning piglets. Furthermore, SEM images showed that LSA could significantly improve the intestinal villi structure and protect the intestinal barrier. Taken together, our findings indicated that LSA effectively reduced intestinal damage, protected the tight junction, reduced intestinal permeability, enhanced the barrier function of the intestinal tract, and improved the intestinal stress response in weaning piglets.

The intestine is an organ that can produce a lot of free radicals. A very small number of free radicals are used by the body, while others are eliminated by antioxidant enzymes and endogenous and exogenous antioxidant systems [8]. The production and elimination of free radicals are in a dynamic balance and can be maintained at a favorable and harmless level to prevent the occurrence and development of diseases [35]. The gastrointestinal tract mainly plays an antioxidant role through antioxidant enzymes, including GSH-Px, SOD, and CAT, to remove free radicals from the body and to prevent oxidative damage [8,34]. Weaning is a stressful process associated with intestinal disorders, which may lead to diarrhea and increased disease susceptibility [36]. Oxidative stress induced by weaning is responsible for intestinal mucosal injury [37]. However, the mechanisms underlying weaning stress-induced gastrointestinal disease are unclear. Excessive reactive oxygen and reactive nitrogen species production and changes in the antioxidant defense systems (SOD, CAT, GSH-Px, and T-AOC) may result in oxidative stress [11,38]. A previous study demonstrated that T-AOC, SOD, and GSH-Px activities in piglets were improved when selenium was added in the form of organic selenium, instead of inorganic selenium, in the diet [39]. In this study, LSA significantly enhanced serum GSH-Px, SOD, CAT, and T-AOC levels; decreased MDA levels; and significantly enhanced the expression of the antioxidant genes (GSH-Px1/2, SOD1/2, CAT) in the ileum. Taken together, our findings indicated that LSA significantly enhanced the intestinal antioxidant capacity and reduced the oxidative injury induced by weaning stress.

Activation of Nrf2 leads to the induction of various cytoprotective proteins, such as Heme oxygenase-1 (HO-1) and NAD(P)H quinine oxidoreductase 1 (NQO1) [40,41]. Transcription factor Nrf2 binds to the antioxidant response element (ARE), which promotes the genes expression of phase II detoxification enzyme, antioxidant enzyme, molecular chaperone, and anti-inflammatory factor, and protects the normal function of tissue cells [42]. In the present study, LSA remarkably increased the gene expression levels of Nrf2 and its downstream phase II detoxification enzymes NQO1 and HO-1. Moreover, mRNA expression levels of antioxidants (GSH-Px, SOD, CAT) in the ileum was consistent with that of Nrf2. These results were in agreement with those of previous studies showing that selenium or selenium compounds can exert cytoprotective effects via activation of Nrf2–ARE signaling pathway [43,44,45]. Our previous study suggested that LSA has good cytoprotective effects against H_2_O_2_-induced oxidative stress in IPEC-1 cells, which may be associated with the activation of the Keap1–Nrf2 signaling pathway [5]. In addition, the study showed that exogenous additives can interfere with intestinal oxidative damage caused by weaning stress, and the mechanism of action is related to Nrf2. Nrf2 is a key factor in cellular oxidative stress. It is regulated mainly by interaction with Keap1, and it binds to the antioxidant responsive element (ARE) to regulate the expression of phase II detoxifying/antioxidant enzymes [46]. When oxidative stress occurs, Nrf2 is released from its inhibitor Keap1 and enters the nucleus. After binding to ARE, Nrf2 promotes the expression of downstream target genes [42]. Nrf2 not only plays an important role in antioxidative stress, anti-inflammatory effects, and cell protection, but it also closely linked with the occurrence and development of various diseases [47]. The mechanism of antioxidant action may be related to the activation of the Nrf2 signaling pathway. To further determine the underlying mechanisms, the effect of LSA on the translocation of Nrf2 and Keap1 was investigated by Western blot analysis in the present study. The results showed that LSA significantly inhibited the expression of Keap1 and increased the expression level of Nrf2. Moreover, the effect of LSA was significantly better than that of inorganic selenium. Our findings suggested that LSA might activate the Nrf2 signaling pathway and promote the transcription of Nrf2 into the nucleus, and enhance the expression of antioxidant enzymes and phase II detoxification enzymes.

## 4. Materials and Methods

### 4.1. Chemicals and Reagents

DAO and d-LA kits were purchased from Nanjing Senbeijia Biological Technology Co., Ltd. (Nanjing, China). Antioxidant kits (SOD, GSH-Px, CAT, MDA, LDH) were purchased from Nanjing Jiancheng Bioengineering Institute (Nanjing, China). 2000 DNA Marker, RNA loading buffer, DNA 6× loading buffer, RIPA lysis buffer, PMSF, BCA kit, BeyoECL Star kit, 5× loading buffer, BSA, non-fat dried milk, and TEMED were obtained from Beyotime Biotechnology (Shanghai, China). Monoclonal antibodies against β-actin were from CST (Cell Signaling Technology, Inc., Boston, MA, USA). Antibody against Nrf2 was purchased from Abcam (Abcam, Cambridge, UK). Monoclonal antibodies against Keap1 were acquired from Santa Cruz (Santa Cruz Biotechnology, Inc., Santa Cruz, CA, USA). All other reagents were of the highest grade or analytical grade. 

The LSA was synthesized in our laboratory and the selenium content in LSA was 17.5 mg/g. The results of UV, IR, NMR analysis showed that the SeO_3_^2−^ is attached to C2 amino and C6 hydroxyl group of amino polysaccharides (data not shown), which was consistent with the findings of the study by Miao et al. [48]. Millipore filter (0.22 μm) and Toxin Eraser endotoxin removal kit were used to remove any contaminants and endotoxins of the LSA. Endotoxin was quantified to be less than 0.1 EU/mL by Chromogenic End-Point Tachypleus Amebocyte Lysate in the stock solution. 

### 4.2. Experimental Animals

All procedures in this study were performed in accordance with the Guide for the Care and Use of Laboratory Animals prepared by the Institutional Animal Care and Use Committee of Zhejiang University. The animals used in this experiment were approved by the principles of the Zhejiang University Animal Care and Use Committee (NO. 2012-0178). Weaning piglets were chosen from Jiaxing Dunhao Farming Co., Ltd, Jiaxing, Zhejiang province, China. One hundred and eighty post-weaning barrows (28 day of age, Duroc × Landrace × Yorkshire) were randomly assigned to 6 treatment groups, as indicated in the Table 2. Each treatment was replicated three times with 10 pigs per replicate. The basal diet was a corn–soybean meal-based diet without selenium supplementation and no antibiotics. All piglets were housed in pens equipped with a fully slatted mesh floor and a nipple water drinker. In addition, they were housed under a dry and ventilated facility and kept under the same temperature (25 ± 2 °C). The piglets were fed a common diet for a 5-day adaptation period. The feeding trial lasted 28 days.

### 4.3. Sample Collection

At the end of the animal experiment, the piglets were subjected to 12 h fasting, all piglets were weighed, and six piglets from each treatment injected with 5% pentobarbital sodium solution (50 mg/kg·BW) to anesthetize. Blood was collected from the anterior vena cava in vacuum blood collection tubes, centrifuged at 3000 rpm at 4 °C for 10 min, and serum was acquired. The serum samples were transferred to 1.5 mL Eppendorf (EP) tubes, respectively, snap frozen in liquid nitrogen, and stored at −80 °C until further analysis. For SEM, after washing in PBS (pH 7.4), the midportion of the jejunum (at least 1 cm^2^) was fixed in 2.5% glutaraldehyde and stored at 4 °C. The ileum mucosa was scraped off with a glass slide. The samples were frozen in liquid nitrogen and stored at −80 °C for analysis.

### 4.4. Determination of Growth Performance and Diarrhea Rate

During the experiment, the feed intake was recorded daily, and the piglets were weighed. The average daily feed intake, average daily gain and ratio of feed/gain were calculated as follows:

average daily feed intake = feed intake/days

average daily gain = (final weight − weaning weight)/days

feed/gain ratio = average daily feed intake/average daily gain

The degree of diarrhea was indicated by the diarrhea index, and a score of 4 can be determined as diarrhea. The specific scores are shown in Table 3. The incidence of diarrhea (%) = the total number piglets with diarrhea over the period divided by the total number of piglets multiplied by the duration of the feeding trial. 

### 4.5. Histopathological Observation

At the end of animal experiment, approximately 1 cm of the jejunum was excised. According to the requirements of SEM, the jejunum was quickly fixed pre-cooled using 2.5% glutaraldehyde. The samples were sent to Zhejiang University for analysis.

### 4.6. Determination of DAO and d-LA in Serum

The serum samples were taken out and thawed. The contents of DAO and d-LA in serum were measured using Enzyme-Linked Immunosorbent (ELISA) Assay Kit. The specific procedure was operated according to the manufacturer’s instructions.

### 4.7. Determination of Antioxidant Indices in Serum

The activities of T-AOC, SOD, GSH-Px, CAT, and MDA test kits were acquired from Nanjing Jiancheng Bioengineering Institute (Nanjing, China). These tests were performed according to the manufacturer’s instructions.

### 4.8. Quantitative Real-Time Polymerase Chain Reaction (RT-PCR)

The mRNA expression level of antioxidant genes (GSH - Px1/2, SOD1/2, CAT, Nrf2, NQO1, HO-1) and connexin genes (ZO-1, Occludin) were measured using quantitative Real-Time polymerase chain reaction (RT-PCR) as described previously [5]. Total RNA was extracted from in the ileum using ice-cold TRIzol reagent (15596026, Thermo Fisher Scientific, Inc., Waltham, MA, USA). The quality and concentration of the extracted total RNA were determined using NanoPhotometer (P300, Implen GmbH, Munich, Germany) by measuring the absorbance at 230, 260 and 280 nm, and by determining the A260/A280 and A260/A230 ratios. The first strand of cDNA was synthesized with using PrimeScript™ II 1st Strand cDNA Synthesis Kit (6210A, Takara Bio, Inc., Ohtsu, Japan) according to the manufacturer’s instructions. The PCR primers were designed using Primer 5 software and from Sangon Biotech Co., Ltd (Shanghai, China) (Table 4). Quantitative polymerase chain reaction (PCR) was carried out using Applied Biosystems ViiA™ 7 Real-Time PCR system. For each PCR reaction, the total volume of 20 μL contained 10.0 μL of SYBR Green qPCR mix, 2 μL cDNA, 0.4 μL of ROX II, 6.8 μL of double distilled water, and 0.4 μL of primers (10 μM forward and 10 μM reverse). RT-PCR reactions were carried out according to the following protocol: pre-denaturation at 95 °C for 30 s, then 40 cycles at 95 °C for 5 s and 65 °C for 34 s. The cycle threshold (CT) values of each gene were normalized with β-actin. β-Actin gene was selected as reference gene for 2^−∆∆Ct^ analysis. 

### 4.9. Western Blot Analysis

Western blot analysis of protein was carried out as described previously [5]. In brief, around 100 mg of intestinal mucosa was placed in liquid nitrogen, ground thoroughly, and collected in a sterile 1.5 mL EP tube. Then, 1 mL of ice-cold RIPA lysis buffer was added into the tube and samples were fully lysed by incubating for 15 min on ice. Protein concentration was measured by BCA method. Proteins were denatured using a loading buffer with the anionic denaturing detergent sodium dodecyl sulfate (SDS), and boiled at 95–100 °C for 5 min. Equal amounts of protein were separated by SDS-PAGE (10%), transferred to 0.45 μm polyvinylidene difluoride (PVDF) membranes, and then blocked with 5% non-fat milk in Tris buffered saline with Tween 20 for 1 h, and incubated with primary antibodies at 4 °C overnight. Subsequently, the membranes were washed three times for 5 min with TBST. Then, the membranes were incubated with secondary antibody (HRP-conjugated secondary antibody) for 1–2 h. ECL reagents were added for chemiluminescent imaging. The bound antibodies were visualized using an enhanced chemiluminescent detection system FluorChem FC3 (ProteinSimple, Santa Clara, CA, USA) and the density of bands were measured using AlphaView software (3.4.0.0, ProteinSimple, Santa Clara, CA, USA). The intensity ratio of proteins was determined relative to β-actin. Each sample was replicated three times. 

### 4.10. Statistical Analysis

All data were evaluated with SPSS17.0 software. Data were presented as mean ± standard error (SEM). The statistical significance was analyzed by one-way analysis of variance (ANOVA) and Tukey’s test. *P* values less than 0.05 were considered as statistically significant.

## 5. Conclusions

The present study indicated that LSA could promote the growth of weaning piglets via increasing the average daily gain and feed/gain, and remarkably reducing the diarrhea rate. LSA effectively reduced intestinal damage, and protected the integrity of intestinal structure and permeability through decreasing the levels of DAO and d-LA. Moreover, LSA significantly enhanced the antioxidant capacity and reduced oxidative stress through increasing the antioxidant index levels, such as through T-AOC, GSH-Px, SOD, and CAT in weaning piglets, while significantly increasing GSH-Px-1, GSH-Px-2, SOD-1, SOD-2, CAT, Nrf2, HO-1, and NQO1 gene expression in intestinal mucosa. In addition, LSA could activate the Nrf2 signaling pathway by downregulating the expression of Keap1 and upregulating the expression of Nrf2. Collectively, LSA may be used as an active substance for the prevention and management of gastrointestinal disorders by weaning stress in infants.

## Figures and Tables

**Table 1:** Effects of low molecular weight seleno-aminopolysaccharide (LSA) on growth performance *.

Item	C	S	S+	LSA1	LSA2	LSA3
Initial weight (kg)	8.28 ± 0.09	8.28 ± 0.12	8.35 ± 0.17	8.33 ± 0.12	8.30 ± 0.15	8.31 ± 0.13
Final weight (kg)	19.36 ± 0.87 ^b^	19.50 ± 0.81 ^b^	19.49 ± 0.93 ^b^	20.29 ± 0.90 ^a^	20.42 ± 1.07 ^a^	19.35 ± 0.90 ^b^
Average daily gain (g)	395.89 ± 31.3 ^b^	400.70 ± 30.10 ^b^	398.07 ± 35.59 ^b^	427.03 ± 31.06 ^a^	432.90 ± 37.51 ^a^	394.32 ± 33.91 ^b^
Average daily feed intake (g)	662.15 ± 10.28	648.57 ± 8.84	651.31 ± 16.87	650.36 ± 5.71	666.79 ± 8.89	646.55 ± 8.47
Feed/gain ratio	1.67 ± 0.05 ^a^	1.62 ± 0.04 ^a^	1.64 ± 0.05 ^a^	1.52 ± 0.03 ^b^	1.54 ± 0.03 ^b^	1.64 ± 0.04 ^a^

Notes. C (control), S (sodium selenite), S+ (sodium selenite + low molecular weight aminopolysaccharide (LA)), LSA1: basal diet + 17.15 mg/kg LSA (0.3 mg/kg Se), LSA2: basal diet + 34.30 mg/kg LSA (0.3 mg/kg Se), LSA3: basal diet + 51.45 mg/kg LSA (0.3 mg/kg Se). * In the same line, values with different letters (a, b, c, d, e) were significantly different (*p* < 0.05).

**Table 2:** Animal grouping *.

Groups	Feed Composition
Control (C)	Basal diet
Na_2_SeO_3_ (S)	Basal diet + 0.3 mg/kg Se (Na_2_SeO_3_)
Na_2_SeO_3_ + LA (S+)	Basal diet + 0.3 mg/kg Se (Na_2_SeO_3_) + 11 mg/kg LA
LSA1	Basal diet + 17.15 mg/kg LSA (0.3 mg/kg Se)
LSA2	Basal diet + 34.30 mg/kg LSA (0.6 mg/kg Se)
LSA3	Basal diet + 51.45 mg/kg LSA (0.9 mg/kg Se)

* Selenium (Se) is measured in form of LSA and sodium selenite.

**Table 3:** Incidence of diarrhea.

Degree of Diarrhea	Fecal Appearance	Water Volume (%)	Score
No diarrhea	Normal, strip, or long strip	<70	1
Mild diarrhea	Soft, basically shaped	70–75	2
Moderate diarrhea	Viscous, not formed, no separation of manure	75–80	3
Severe diarrhea	Liquid, not formed, separation of manure	>80	4

**Table 4:** Real-Time PCR Primers and Conditions.

Gene	Gene Accession Number	Primer Sequence 5′-3′	PCR Product Size (bp)	Tm
SOD1	NM_000454	F: TCCATGTCCATCAGTTTGGA R: CTGCCCAAGTCATCTGGTTT	250	50
SOD2	NM_001322820	F: TGGAGGCCACATCAATCATA R: AGCGGTCAACTTCTCCTTGA	136	59
CAT	NM_214301.2	F: TGTGAACTGTCCCTTCCGTG R: CGTCTGTTCGGGAGCACTAA	124	60
GPx1	NM_201397.2	F: TGGGGAGATCCTGAATTG R: GATAAACTTGGGGTCGGT	184	53
GPx2	NM_001115136.1	F: TCGTGGCTTCCCTTGCAAC R: CCATTCACGTCACACTTCTG	138	61
Nrf2	XM_021075133.1	F: CACCACCTCAGGGTAATA R: GCGGCTTGAATGTTTGTC	125	55
HO-1	NM_001004027.1	F: AGCTGTTTCTGAGCCTCCAA R: CAAGACGGAAACACGAGACA	130	59.2
NQO1	NM_001159613.1	F: CCAGCAGCCCGGCCAATCTG R: AGGTCCGACACGGCGACCTC	160	66.2
ZO-1	NM_001355015	F: CAGCCCGAGGCGTGTTTA R: TGGGAGGATGCTGTTGTCT	146	60
Occludin	NM_214301.2	F: GCTGGAGGAAGACTGGAT R: ATCCGCAGATCCCTTAAC	124	61
β-actin	XM_003124280	F: GATGAGATTGGCATGGCTTT R: CACCTTCACCGTTCCAGTTT	122	60

F, forward; R, reverse.
